# Rapid Quantitative Analysis of Forest Biomass Using Fourier Transform Infrared Spectroscopy and Partial Least Squares Regression

**DOI:** 10.1155/2016/1839598

**Published:** 2016-11-24

**Authors:** Gifty E. Acquah, Brian K. Via, Oladiran O. Fasina, Lori G. Eckhardt

**Affiliations:** ^1^Forest Products Development Center, School of Forestry and Wildlife Sciences, Auburn University, 520 Devall Drive, Auburn, AL 36849, USA; ^2^Center for Bioenergy and Bioproducts, Department of Biosystems Engineering, Auburn University, 350 Mell Street, Auburn, AL 36849, USA; ^3^Forest Health Dynamics Laboratory, School of Forestry and Wildlife Sciences, Auburn University, 602 Duncan Drive, Auburn, AL 36849, USA

## Abstract

Fourier transform infrared reflectance (FTIR) spectroscopy has been used to predict properties of forest logging residue, a very heterogeneous feedstock material. Properties studied included the chemical composition, thermal reactivity, and energy content. The ability to rapidly determine these properties is vital in the optimization of conversion technologies for the successful commercialization of biobased products. Partial least squares regression of first derivative treated FTIR spectra had good correlations with the conventionally measured properties. For the chemical composition, constructed models generally did a better job of predicting the extractives and lignin content than the carbohydrates. In predicting the thermochemical properties, models for volatile matter and fixed carbon performed very well (i.e., *R* ^2^ > 0.80, RPD > 2.0). The effect of reducing the wavenumber range to the fingerprint region for PLS modeling and the relationship between the chemical composition and higher heating value of logging residue were also explored. This study is new and different in that it is the first to use FTIR spectroscopy to quantitatively analyze forest logging residue, an abundant resource that can be used as a feedstock in the emerging low carbon economy. Furthermore, it provides a complete and systematic characterization of this heterogeneous raw material.

## 1. Introduction

Lignocellulosic biomass is the only renewable resource that can be used in the production of biofuels and platform chemicals in addition to bioenergy. As the most abundant carbon neutral resource, using biomass instead of fossil fuels can help mitigate environmental pollution. However, many physical, chemical, and structural factors can hinder the conversion of biomass into fuels and chemicals. A better understanding of the properties of biomass will be important in the allocation of feedstock to the appropriate end use. An ability to determine these properties in a timely manner is also necessary in the optimization of conversion technologies for the successful commercialization of biomass-based products. There is thus a need for high throughput methods and equipment in the monitoring and characterization of the raw feedstock as conventional methods have been laborious and destructive.

Fourier transform infrared reflectance (FTIR) spectroscopy has been used as a powerful analytical tool for the rapid characterization of lignocellulosic biomass. Since FTIR spectroscopy determines the presence of fundamental molecular vibrations that are characteristic of a chemical compound or class of compounds, it has widely been used in the qualitative elucidation of changes in biomass structure during and/or after treatment with processes. For instance, FTIR spectroscopy was used to study trembling aspen extracted with supercritical methanol [1] and also to monitor the physical and chemical changes that result as corn stover undergoes ammonia fiber expansion (AFEX) and iconic liquid (IL) pretreatments [2]. With the advancement of multivariate data analysis, researchers are now coupling FTIR spectroscopy with chemometric methods for rapid quantitative analysis of biomass feedstock. FTIR-based partial least squares (PLS) models were constructed to quantify the monomeric sugars, acetic acid, and 5-hydroxymethyl-2-furfural (HMF) of dilute acid pretreated biomass [3]. The tool has also been used to model the ash, volatile matter, fixed carbon, and higher heating value (HHV) of sweetgum, loblolly pine, and switchgrass torrefied at different temperatures [4]. For some studies on the raw biomass, FTIR spectroscopy was employed in characterizing several agricultural residues and their extractives content [5], in the qualitative analysis of lignin from five timber species [6], and in predicting the chemical composition of hardwoods [7]. FTIR-based models have also been employed for discriminant and classification analysis of biomass feedstocks and plant materials [8–12].

Most of the studies using FTIR spectroscopy were conducted on biomass that has been subjected to some kind of pretreatment; surprisingly, not very much was found on the raw resource.

In the USA, about 1.3 million dry tons of biomass can be sourced annually from forestry (27%) and agricultural (77%) operations, capable of replacing a third of the country's current fuel consumption [13]. Forest biomass includes logging residues, precommercial thinnings, fuel treatments, residues from primary and secondary mill processing, and urban wood wastes. According to Smith et al. (2009) [14], some 68 million dry tons of logging residues are currently produced in the USA, most of which is left onsite. Using logging residue as a raw material will ensure more complete and sustainable utilization of trees. In addition, several studies have shown that the sustainable removal of logging residues can improve forest health, enhance replanting efforts and regeneration, and control forest fires [15–17].

Qualitative and quantitative analysis of biomass with FTIR spectroscopy can be quite precise when materials vary considerably in chemical structure. For example, woody tissue could be easily differentiated from bark resulting in an easy calculation of bark content in aspen and birch [18] and beech could be differentiated from pine due to considerable differences in syringyl and guaiacyl moieties [19]. Such differentiation was less discriminative for the same tree species with tissue obtained from the juvenile and mature wood [20]. However when perturbations such as temperature [4] and chemical treatments [21] are introduced then model robustness for identification and/or concentration determination becomes more superior. The objective of this study was to employ FTIR spectroscopy coupled with partial least squares (PLS) regression to rapidly predict the chemical composition, thermal reactivity, and energy content of logging residue of loblolly pine, the most economically important tree species in the USA. This study attempts to take advantage of the wide differences in bark, needle, and woody tissue chemistry that should allow for easy discrimination and quantification. The accurate estimation of, for instance, the concentration of carbohydrates is important since it is directly proportional to the yield of biofuels and having prior knowledge of the inorganic fraction will enable the anticipation of slagging or the extent to which the calorific value may be impacted.

## 2. Materials and Methods

### 2.1. Materials

Lignocellulosic biomass acquired during harvesting operations on several loblolly pine plantations in southern Alabama, USA, was used for this study. Material comprised whole tree, wood and bark, slash (i.e., limbs and foliage), and clean wood chips. Ten biomass sets were sampled for each of the four biomass types.

### 2.2. Methods

#### 2.2.1. Conventional Laboratory Methods

Conventional standard methods were used to determine the chemical makeup, thermal reactivity, and energy content of biomass.

The chemical composition of biomass samples was determined via wet chemistry. Extractive content was determined as specified in NREL/TP-510-42619 and TAPPI T- 204. 5 g of a test sample ground to pass a 40-mesh screen was extracted in 150 mL of acetone for 6 hours in a Soxhlet Apparatus. Afterwards, the acetone was allowed to evaporate before drying the extract in a vacuum oven at 40°C for 24 hours. Air-dried extractive-free samples were used to determine lignin and carbohydrates following NREL/TP-510-42618. Test samples were first hydrolyzed with 72% sulfuric acid. This primary hydrolysis was carried out at 30°C ± 3°C for an hour. Then, the concentration of acid was diluted to 4% with deionized water and a secondary hydrolysis carried out in an autoclave at 121°C for another hour. Hydrolyzed samples were allowed to cool before filtering through tared glass crucibles. An aliquot of this filtrate was collected to be used for determining the acid-soluble lignin (ASL) and monomeric sugars. The solid residue was thoroughly washed with distilled water and oven dried at 105°C overnight and the final weight used for calculating the acid-insoluble lignin (AIL) content of biomass. The ASL was determined with a UV/vis spectrophotometer immediately after hydrolysis. Total lignin was calculated as the sum of ASL and AIL. The monomeric sugars (i.e., arabinose, glucose, galactose, mannose, and xylose) in test samples were measured using a Bio-Rad Aminex HPX-87P column equipped HPLC. Holocellulose content was calculated as the sum of monomeric sugars; the cellulose content was computed as glucose − ((1/3)*∗*mannose) and the difference between holocellulose and cellulose designated as hemicelluloses.

Bomb calorimetry, as specified in ASTM D5865, was used in calorific value determination, whereas proximate analysis was conducted following NREL/TP-510-42622 (for ash content) and CEN/TS 15148 (for volatile matter content).

Analysis of Variance (ANOVA) followed by Tukey pairwise comparison tests between the four biomass types (*α* = 0.05) was performed using the R Stats Package. Duplicate experiments were run for each test sample.

#### 2.2.2. Collection of Spectra

Mid infrared spectra were collected over a wavenumber range of 4000 cm^−1^ to 650 cm^−1^ using a PerkinElmer Spectrum 400 FT-IR/FT-NIR spectrometer equipped with a diamond crystal attenuated total reflectance device (i.e., ATR-FTIR) and a torque knob to ensure that consistent pressure is applied to samples during spectra collection. Prior to spectra acquisition, samples were ground to pass an 80-mesh screen to improve model properties through light scatter reduction and oven dried at 40°C for 4 hours. Each test sample was placed on the diamond plate, a pressure of 70 ± 2 psi applied using the torque knob, and then scanned thirty-two times at a resolution of 4 cm^−1^. The average of the thirty-two spectra was corrected for background absorbance by subtracting the spectrum of the empty ATR crystal and used for analysis.

#### 2.2.3. Partial Least Squares (PLS) Regression

 PLS regression is a statistical technique for developing predictive models of multivariate data that otherwise have high collinearity. The iterative NIPALS algorithm used extracted successive linear combinations of the predictors (called factors or latent vectors) such that variations in both predictors (i.e., MIR spectra) and responses (i.e., property under study) were optimally accounted for. A more detailed description of the procedure can be found elsewhere [22].

PLS models were developed with PerkinElmer's Spectrum Quant^+^ software using spectra of the full mid infrared region (i.e., 4000–650 cm^−1^) as well as the fingerprint region (1800–650 cm^−1^). Spectra were pretreated with derivatives (1st order 5-point) for baseline correction and also to help reduce nonlinearity and multicollinearity among variables. Second derivatives were not used due to the generally lower signal to noise ratio. Both predictors and responses were mean centered prior to modeling. Due to the relatively small sample size (*n* = 40), the leave-one-out cross validation modeling method was employed. In this technique, for each run, 39 out of the 40 samples are used as training dataset for calibrating a model that is used to predict the 1-sample test dataset. This is iterated forty times until all samples are used as independent single-element test datasets.

Developed models were evaluated using such statistics as the standard error of calibration (SEC), standard error of cross validation (SECV), coefficient of determination (*R* ^2^), and ratio of performance to deviation (RPD).

## 3. Results and Discussion

### 3.1. FTIR Spectra

**Figure 1:**
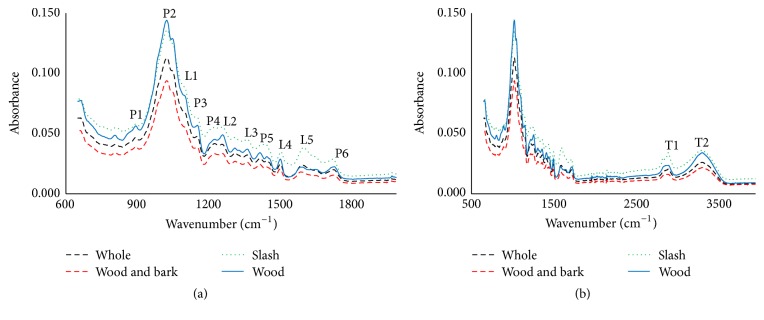
FTIR spectra of the different types of forest biomass. (a) Fingerprint region; (b) full MIR range.

MIR spectra characteristics of the four biomass sets understudied are presented in Figure 1. Even though this region encompasses the 4000 to 650 cm^−1^ wavenumber range, the fingerprint region (1800 to 650 cm^−1^) is usually of particular interest because it contains the most spectral information pertaining to the molecular/chemical composition of a material (Figure 1(a)). In the literature, several bands have been linked to carbohydrates due to their associated functional groups. Within the fingerprint region, peaks that result due to the polysaccharides include (P1) 897 cm^−1^ and (P2) 1030 cm^−1^ from the C-H deformation in cellulose and C-O stretch in polysaccharides, respectively, (P3) 1157 cm^−1^ from C-O-C vibration, (P4) 1239 cm^−1^ from C-O stretch and O-H in plane in polysaccharides, (P5) 1465 cm^−1^ from C-H deformation, and (P6) 1740 cm^−1^ from the C=O stretching of unconjugated ketones mostly in the hemicelluloses. In the case of lignin, the peak at (L1) 1122 cm^−1^ occurs due to aromatic skeletal and C-O stretch. Guaiacyl ring breathing with C-O stretching causes a peak to rise at (L2) 1270 cm^−1^ and syringyl ring breathing creates the peak at (L3) 1365 cm^−1^. The strong peak at (L4) 1505 cm^−1^ is attributed to the C=C stretch characteristic of aromatic skeletal compounds in lignin and extractives. Outside the fingerprint range (Figure 1(b)), the peaks occurring at (T1) 2935 cm^−1^ have been associated with the bending and stretching of C-H, as well as its aromatic ring vibration in lignin, whereas that at (T2) 3345 cm^−1^ has been assigned to bonded O-H [1, 23–26].

The four biomass types followed a similar absorbance pattern in the mid infrared region. Slash generally had the highest absorbance values, followed by wood, whole, and then wood and bark. The spectra of slash had prominent peaks at (L5) 1635 cm^−1^ and (T1) 2935 cm^−1^ compared to the other biomass types. The former has been attributed to C-O stretching of conjugated or aromatic ketones and/or C=O stretching vibration in flavones, and the latter results from the aromatic ring vibration in lignin [27–29]. These high peaks could thus be explained by the significantly high contents of extractives and lignin in slash Figure 1.

These assignments provide some insight into the chemical moieties present in the different biomass types. However, the overlapping peaks make it challenging to tease out subtle difference by simple visual inspection sometimes [30]. As such, the application of multivariate data analytical techniques to the spectra of lignocellulosic biomass helps to extract relevant information and structure spectra and conventionally acquired chemical data into empirical mathematical models that are capable of predicting properties of future measurements and even other properties that are not directly measurable [31]. Scatter, stray light, path length variation, inconsistency in instrument response, and random noise can cause interferences such as baseline shifts, vertical displacements, and nonuniform slope in infrared spectra. Pretreatment methods including standard normal variate (SNV) transformation, multiplicative scatter correction (MSC), derivatives, and orthogonal signal correction (OSC) are therefore usually used to minimize, standardize, or even eliminate the impacts of these interferences on IR spectra before multivariate data analysis to improve the robustness of calibration models. In this study, the first derivatives of spectra were used to reduce baseline offsets and improve the resolution of overlapping peaks [32].

### 3.2. PLS Modeling of the Chemical Composition of Forest Logging Residue

**Figure 2:**
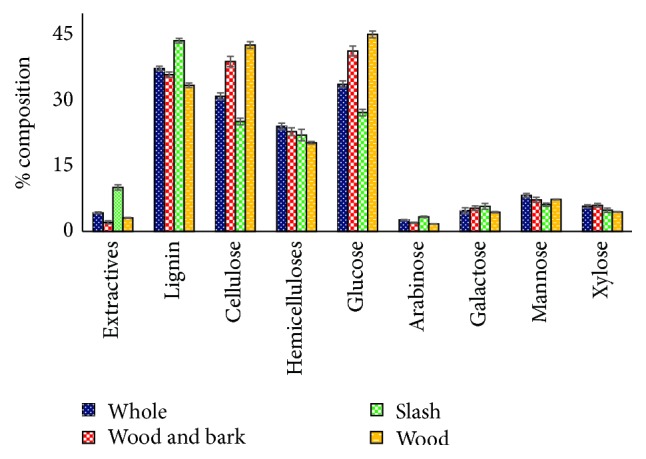
Descriptive statistics of the chemical composition of forest logging residue. NB: bars represent ± standard error.

The chemical composition of forest logging residue determined via conventional methods is summarized in Figure 2. Some significant differences (*α* = 0.05) were noted among the four biomass types. The mean concentration of extractives ranged from a low of 2% for wood and bark to a high of 10% for slash. The percentage of glucose was significantly lower in slash (27%) and whole (34%) compared to wood and bark (41%) and wood (45%). This pattern was unsurprisingly followed by the amount of cellulose in the four types of forest logging residue. Whole had the highest amount of hemicelluloses, and this was statistically similar to the concentrations found in slash and wood and bark.

Using spectra as the independent variable and a measured property as the dependent variable, all forty biomass samples were employed in the calibration and cross validation of PLS predictive models. Models were developed using raw or 1st-derivative spectra of first entire MIR range and then the fingerprint region. As is generally the case, models built with 1st-derivative treated spectra have better predictive capabilities compared to those calibrated with untreated spectra; thus only results of the former are presented in this paper (Table 1). Optimum models were chosen as those that used a lesser number of latent variables (LVs) to produce smaller error values. Another consideration in final model selection was a small difference between the SEC and SECV. The SECV (which is a better measure of a model's predicting ability of future unknowns) is usually larger than the SEC (a statistic that evaluates how precisely the regression line fits the training data) because it also takes into consideration how much worse a model performs on independent test data not originally included in model calibration. However, the SECV ideally should be no greater than 1.3 times the SEC [33]. A big difference in SEC and SECV results when calibration models do a poor job of predicting the property under study for samples that were used in cross validation.

Two or three LVs were used in the development of models that had *R* ^2^ values ranging from a low of 0.64 for galactose to a high of 0.93 for extractives (Table 1). Although *R* ^2^ is an indicator of a good model (when greater than 0.5), it was not used as the sole assessor of models because it usually has a direct relationship with the number of LVs used in model development. When more LVs are added in calibration, a model continues to fit random errors until every source of variation is accounted for in the training data [34]. The RPD, which is computed as the ratio of standard deviation of the validation set to the standard error of prediction (SEP), was used to compare the predictive ability of models. Except for galactose, xylose, and hemicelluloses, the RPD values of models developed to predict the chemical components of forest logging residue fell within the preliminary screening criteria (i.e., 1.5–2.5) [35]. The model for extractives was the most robust, having an RPD of 2.3.

PLS modeling of MIR spectra did a better job of predicting the extractives and lignin content of loblolly pine logging residue compared to the structural carbohydrates and their associated monomeric sugars in this study. The best performing models were for glucose and arabinose both of which had *R* ^2^ of 0.77 and RPD of 1.6, whereas the worst performing models were for galactose and mannose and the other two hexoses. Poor prediction of monomeric sugars has been attributed to similar conformation of sugars that only differ in the orientation of some hydroxyl groups. In a previous study [22], galactose, mannose, xylose, and consequently hemicelluloses were also poorly predicted by near infrared- (NIR-) based PLS models. Similarly poor performing models were obtained by [36] for galactose (*R* ^2^ = 0.11, RPD = 0.8) and hemicelluloses (*R* ^2^ = 0.30, RPD = 1.0). Since FTIR spectroscopy detects fundamental molecular vibrations as opposed to the overlapping and usually weaker combination bands in NIR, PLS models developed in this study were expected to do a better job of predicting the monomeric sugar content of forest logging residue, but this unfortunately was not the case.

The entire MIR range and fingerprint region were also used to model the lignin, cellulose, and extractives of wood samples including* Scots pine*, Sitka spruce, and tropical hardwoods from Ghana [28]. The authors reported the performance statistics of PLS models as follows: cellulose: *R* ^2^ = 0.65, SEC = 1.8, and SEP = 3.3; lignin: *R* ^2^ = 0.65, SEC = 1.8, and SEP = 3.3; and extractives: *R* ^2^ = 0.93, SEC = 0.3, and SEP = 0.4. The seeming trend of infrared-based PLS models predicting the lignin and extractives of biomass relatively better than the polysaccharides was noted in this study also and again in [37] when both diffuse reflectance (DRIFT) and transmission FTIR spectra were used in PLS modeling. A possible explanation of this trend could be the distinctive chemical structures of lignin and extractives, as opposed to the relative abundance of carbohydrates that have similar molecular makeup. Another study that quantitatively characterized the chemical composition of untreated wood was by [38]. The researchers developed DRIFT-PLS models for lignin (*R* ^2^ = 0.66, SEP = 1), extractives (*R* ^2^ = 0.97, SEP = 0.9), arabinose (*R* ^2^ = 0.79, SEP = 0.1), galactose (*R* ^2^ = 0.80, SEP = 0.3), glucose (*R* ^2^ = 0.57, SEP = 1.7), mannose (*R* ^2^ = 0.63, SEP = 0.8), and xylose (*R* ^2^ = 0.73, SEP = 0.5). The standard deviations of the training data and prediction errors of monomeric sugars reported by the authors were low, even though the *R* ^2^ values are similar compared to what was obtained in current study.

For a fairer comparison of model performance, the same number of LVs that were retained as optimum for full spectra (4000–650 cm^−1^) models was used in developing reduced spectra (1800–650 cm^−1^) models. Reducing the wavenumber range to the fingerprint region did not adversely affect the performance of PLS models (Table 1). In fact, this generally decreased the errors associated with cross validation (employed as the SEP in current study) and improved RPD values for all models except that for lignin (full: SECV = 2.02, RPD = 2.06; reduced: SECV = 2.04, RPD = 2.04) and galactose (full: SECV = 1.87, RPD = 0.93; reduced: SECV = 2.05, RPD = 0.85) (Table 1). Lowered SECV and improved RPD values are an indication that a model's predictive capability is reduced when irrelevant wavenumbers are included in model construction.

**Figure 3:**
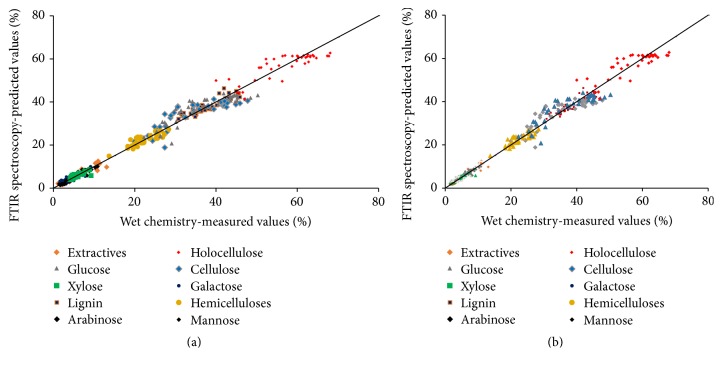
A regression plot of wet chemistry-measured versus FTIR-predicted values for chemical composition. (a) Modeled with full spectra; (b) modeled with fingerprint region.

The relationships between laboratory reference data and FTIR-predicted chemical constituents are presented in Figure 3.

### 3.3. PLS Modeling of Thermal Reactivity and Energy Content of Forest Logging Residue

**Figure 4:**
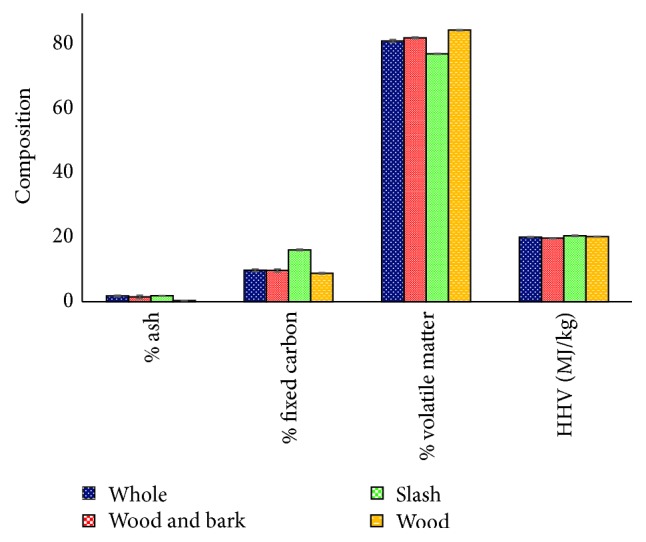
Descriptive statistics of the thermal reactivity and energy content of forest logging residue. NB: bars represent ± standard error.

Summary statistics from proximate analysis and bomb calorimetry are presented in Figure 4. Ash content was significantly lower in wood compared to the other three biomass types as expected. In contrast, wood had the highest amount of volatile matter. Fixed carbon ranged from a low of 8.9% in wood to a high of 16.2% in slash. Among the four biomass types, whole and wood and bark samples were more similar in their thermal reactivity and energy content. The higher heating value (HHV) which is the maximum amount of energy that can be potentially recovered when fuel is completely combusted under adiabatic conditions ranged from 19.8 (MJ/kg) to 20.6 (MJ/kg) for loblolly pine logging residue.

Two or three LVs were used in PLS modeling of the full or reduced MIR spectra. Fit statistics of cross validated models calibrated with the 1st derivative of spectra are presented in Table 2.

For the reduced spectra of volatile matter content, two LVs gave the lowest values of SEC (1.03%) and SECV (1.15) falling within the ideal difference range. This optimized model had *R* ^2^ of 0.88 and an RPD of 2.3. This was a 6% improvement over the RPD value of the model developed using the full MIR range. Similarly, utilizing the fingerprint region slightly improved the RPD value of the model for predicting percent fixed carbon. Correlations of ash content with spectra data were quite high, although the RPD values were less than 1. Unlike for the organic components of forest biomass, developing models with the reduced spectra for the inorganic ash increased both the SEC and SECV and reduced the *R* ^2^ and RPD values. Poor performance was also reported for full spectra FTIR-based PLS models constructed to predict the ash content of two energy crops (SEC = 1.02, SECV = 1.08, and *R* ^2^ = 0.48) [18]. However, the authors in [4] were able to better model the ash content of torrefied biomass. Simple monoatomic inorganic compounds do not produce any vibrations in the mid (or near) infrared region. However, these form complexes with organic species to produce characteristic bands. As such, FTIR and NIR spectroscopy have been capable of quantitative and qualitative analysis of the ash in biomass, polymers, and so forth [30].

Energy content is known to be influenced by the chemical composition of biomass. Lignin, (which can have as much as twice the calorific value of the carbohydrates) and extractives are mostly credited for this [39]. Consequently, FTIR spectroscopy which is sensitive to chemical signals has been used to model the HHV of biomass. PLS models constructed in this study to predict the HHV however did not perform very well. The *R* ^2^ values of models were 0.64 and 0.54 and RPD values were 1.03 and 1.23 using the full and reduced spectra, respectively (Table 2).

**Figure 5:**
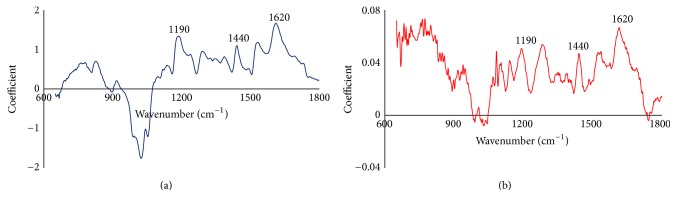
Regression spectra showing some common wavenumbers that made significant contribution to the modeling of extractives (%) (a) and HHV (MJ/kg) (b).

Simple linear regression models were developed to explore the relationship between the chemical composition of forest logging residue and HHV. Only the extractive content had some meaningful linear correlation with HHV (*R* ^2^ = 0.31, *p* value < 0.05), suggesting that the correlation between FTIR spectra and HHV is a secondary function of the correlation between the extractives and spectra. There have been conflicting reports in the literature about how especially lignin correlates with energy content [40, 41]. Comparing the regression spectra of extractives to that of HHV showed some common wavenumbers/peaks that made significant contributions to the modeling of the two properties, supporting results from the regression analysis (Figure 5). Peaks were noted at 1620 cm^−1^ (skeletal aromatic C=C in plane vibration), 1440 cm^−1^ (C-O stretching, plus OH deformation of carboxylic acids or C-C stretching of aliphatic aldehydes), and 1190 cm^−1^ (C-O stretching of higher esters) [42]. However, unlike for the extractives, the peaks occurring in the regression spectrum of HHV could not account for as much of its variation, thus, the bad prediction performance of this model.

**Figure 6:**
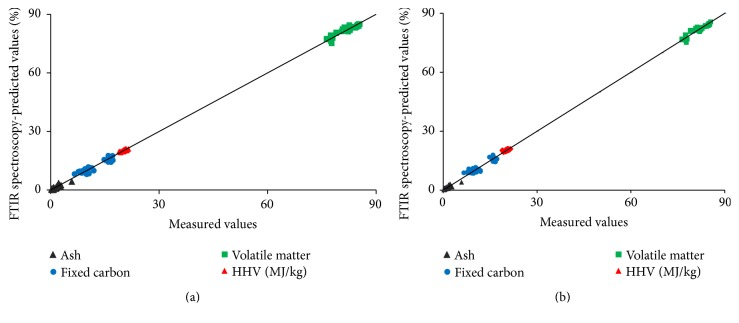
Regression plot of measured versus FTIR-predicted values for thermal reactivity and energy content. (a) Modeled with full spectra; (b) modeled with fingerprint region. Percent except for HHV.

A scatter plot of how FTIR-based PLS models predicted the thermal reactivity and HHV as compared to results determined via proximate analysis and bomb calorimetry is presented in Figure 6.

## 4. Conclusions

FTIR spectra of forest logging residue made up of whole tree, wood and bark, slash, and wood were acquired and related to the chemical and thermal reactivity and energy content of the biomass. PLS models were developed with the raw and 1st derivative of spectra spanning the entire MIR region or the fingerprint region. For chemical composition, developed models generally did a better job of predicting the extractives and lignin content than the carbohydrates; for the thermochemical properties, models for volatile matter and fixed carbon performed very well (i.e., *R* ^2^ > 0.80, RPD > 2.0). Reducing the wavenumber range to the fingerprint region for PLS modeling did not compromise the predictive ability of models. In fact, this mostly reduced the errors associated with prediction and improved the RPD values.

This study demonstrated that the chemical and thermochemical properties of forest logging residue can be predicted with FTIR spectroscopy coupled with PLS. The accuracies of prediction models constructed for this very heterogeneous biomass feedstock were comparable to that measured via lengthy and laborious conventional methods. The suite of important biomass properties understudied was predicted from a single FTIR spectrum without having to do any extra work for each of the properties. Thus FTIR spectroscopy can be employed as a high throughput tool for monitoring and characterizing this largely untapped resource to optimize processes in biorefineries that will depend on logging residues as new markets emerge and conversion technologies advance in the low carbon bioeconomy.

## Figures and Tables

**Table 1:** Performance evaluation of PLS models developed using 1st-derivative treated spectra of the full (i) and fingerprint (ii) regions for predicting chemical composition.

Constituent (%)	Extractives	Lignin	Cellulose	Hemicelluloses	Glucose	Arabinose	Galactose	Mannose	Xylose	Holocellulose
LVs										
(i)	2	2	2	3	2	2	3	3	2	2
(ii)	2	2	2	3	2	2	3	3	2	2
SEC										
(i)	0.93	1.58	3.89	1.32	3.71	0.36	1.09	0.85	0.74	3.92
(ii)	1.03	1.77	4.04	1.61	3.88	0.4	1.03	0.82	0.76	4.25
SECV										
(i)	1.4	2.02	5.1	3.58	4.6	0.46	1.87	1.87	1.13	5.05
(ii)	1.18	2.04	4.58	3.46	4.4	0.46	2.05	1.84	1.06	4.79
*R* ^2^										
(i)	0.93	0.86	0.74	0.82	0.77	0.77	0.64	0.71	0.7	0.73
(ii)	0.91	0.83	0.72	0.74	0.75	0.72	0.67	0.73	0.68	0.68
RPD										
(i)	2.34	2.06	1.46	0.85	1.63	1.57	0.93	0.81	1.16	1.45
(ii)	2.83	2.04	1.61	0.87	1.7	1.6	0.85	0.84	1.24	1.53

**Table 2:** Performance evaluation of PLS models developed using 1st-derivative treated spectra of the full (i) and fingerprint (ii) regions for predicting thermal reactivity and energy content.

Constituent	Ash (%)	Fixed carbon (%)	Volatile matter (%)	HHV (MJ/kg)
LVs				
(i)	3	2	2	2
(ii)	3	2	2	2
SEC				
(i)	0.49	1.26	1.07	0.34
(ii)	0.6	1.35	1.03	0.38
SECV				
(i)	1.07	1.6	1.31	0.53
(ii)	1.09	1.54	1.15	0.44
*R* ^2^				
(i)	0.8	0.85	0.87	0.64
(ii)	0.7	0.83	0.88	0.54
RPD				
(i)	0.98	1.96	2.17	1.03
(ii)	0.96	2.04	2.31	1.23
